# A backward-mode optical-resolution photoacoustic microscope for 3D imaging using a planar Fabry-Pérot sensor

**DOI:** 10.1016/j.pacs.2021.100293

**Published:** 2021-08-10

**Authors:** Elisabeth Baumann, Ulrike Pohle, Edward Zhang, Thomas Allen, Claus Villringer, Silvio Pulwer, Holger Gerhardt, Jan Laufer

**Affiliations:** a Integrative Vascular Biology Laboratory, Max-Delbrück Center for Molecular Medicine in the Helmholtz Association (MDC), Robert-Rössle-Strasse 10, 13125, Berlin, Germany; b Charité – Universitätsmedizin Berlin, Charitéplatz 1, 10117, Berlin, Germany; c Institut für Physik, Martin-Luther-Universität Halle-Wittenberg, Von-danckelmann-platz 3, 06120, Halle (Saale), Germany; d Department of Medical Physics and Biomedical Engineering, University College London, Gower Street, WC1E 6BT, UK; e University of Applied Sciences Wildau, Hochschulring 1, 15745, Wildau, Germany; f DZHK (German Center for Cardiovascular Research), Partner site, Potsdamer Str. 58, 10785, Berlin, Germany; g Berlin Institute of Health (BIH), Anna-Louisa-Karsch-Straβe 2, 10178, Berlin, Germany

**Keywords:** Optical-resolution photoacoustic microscopy, Backward-mode imaging, Dual-wavelength, Continuous scanning, *In vivo* imaging, Optical ultrasound sensing, Planar Fabry-Pérot interferometer

## Abstract

Optical-resolution photoacoustic microscopy (OR-PAM) combines high spatial resolution and strong absorption-based contrast in tissue, which has enabled structural and spectroscopic imaging of endogenous chromophores, primarily hemoglobin. Conventional piezoelectric ultrasound transducers are typically placed far away from the photoacoustic source due to their opacity, which reduces acoustic sensitivity. Optical ultrasound sensors are an alternative as their transparency allows them to be positioned close to the sample with minimal source-detector distances. In this work, a backward-mode OR-PAM system based on a planar Fabry-Pérot ultrasound sensor and coaxially aligned excitation and interrogation beams was developed. Two 3D imaging modes, using raster-scanning for enhanced image quality and continuous-scanning for fast imaging, were implemented and tested on a leaf skeleton phantom. In fast imaging mode, a scan-rate of 100,000 A-lines/s was achieved. 3D images of a zebrafish embryo were acquired *in vivo in raster-scanning mode.* The transparency of the FP sensor in the visible and near-infrared wavelength region makes it suitable for combined functional and molecular imaging applications using OR-PAM and multi-photon fluorescence microscopy.

## 1 Introduction

Optical-resolution photoacoustic microscopy (OR-PAM) provides high-resolution images of biological tissue *in vivo* from which structural and functional information can be obtained. To generate photoacoustic (PA) waves, the output of a pulsed laser is focussed into superficial regions of the tissue (<600−1000 μm) where the absorption of the optical energy by tissue chromophores, such as hemoglobin, results in a localized pressure rise. The initial pressure distribution relaxes by emitting a broadband ultrasonic wave, the amplitude of which is considered proportional to the absorbed energy density, *i.e.* the product of local absorption coefficient and the fluence. To acquire PA image data sets, the focused excitation beam is raster-scanned across the sample and time-resolved photoacoustic signals are acquired at each point. Using the speed of sound in tissue, the time axis is converted to depth, which results in 3D images that show the spatial distribution of the optical absorption, and hence the tissue chromophores. For superficial depths, the lateral resolution is primarily determined by the diffraction limit with typical resolutions of several μm [1,2]. Special techniques have also been shown to increase the resolution to the sub-micron range [[3], [4], [5]].

Due to the strong contrast provided by oxy- (HbO_2_) and deoxyhemoglobin (HHb) at excitation wavelengths in the visible part of the optical spectrum, OR-PAM images typically show the vasculature including capillaries and single red blood cells. By acquiring images at multiple excitation wavelengths and by exploiting the difference in the absorption spectra of HbO_2_ and HHb, OR-PAM can be used to obtain images of physiological parameters, such as blood oxygen saturation, flow, and metabolic rate [[2], [3], [4], [5], [6], [7], [8], [9], [10]], which is of interest in preclinical functional imaging studies of the vasculature [[1], [2], [3], [4], [5], [6], [7], [8], [9],^11^,^12^].

OR-PAM systems that operate in backward-mode, where PA signals are excited and detected on the same side of the sample are advantageous for many *in vivo* applications as they enable larger animals or organs to be imaged. In addition, the acoustic detection sensitivity may be increased by minimizing the source-detector distance [13]. For backward-mode configurations, conventional piezoelectric ultrasound detectors have disadvantages because their opacity requires them to be placed far from the source. While these limitation may be addressed using transparent piezoelectric transducers [[14], [15], [16], [17], [18]], their frequency response is strongly resonant. The development of optical ultrasound sensors offers an alternative as they can be designed to be transparent to the excitation wavelength and therefore be positioned near the PA source. They also typically exhibit high acoustic sensitivity over a broad frequency range [19] and have the potential to be combined with other high-resolution optical imaging methods, such as optical coherence tomography and fluorescence microscopy [[20], [21], [22], [23], [24]]. 3D backward-mode OR-PAM using optical ultrasound detection has been demonstrated using a variety of techniques such as polarization-dependent optical reflection imaging [25], π-shifted Fibre-Bragg grating sensors [26], probe beam deflection [27], and interferometric approaches, such as micro-ring resonators [28,29] and Fabry-Pérot (FP) sensors and probes [[30], [31], [32], [33], [34], [35], [36], [37], [38]]. Transparent FP sensors are particularly attractive for OR-PAM since they can be placed adjacent to the sample with minimal source-detector distances. Also, they combine advantageous properties, such as high acoustic sensitivity, small active element sizes (tens of μm), and a broadband frequency response [13,32]. Different sensor designs are currently used for PA imaging, such as planar and plano-concave cavities. The latter was shown to result in exceptionally high finesse and hence acoustic sensitivity [33,34] and was incorporated as an optical fiber FP sensor in OR-PAM systems [36,37]. The sensor is typically in a fixed position outside to the scan area, which can adversely affect the conversion of time-of-flight information of PA signals to depth with uniform vertical resolution if the source-sensor distances and detection angles are large. Planar FP sensors, which have so far primarily been used for PA tomography [13,20,21,32,[39], [40], [41], [42]], offer several advantageous properties for OR-PAM, such as high acoustic sensitivity at high frequenies (> 100 MHz) for high spatial resolution [13,32] and an omnidirectional response [32,43,44]. Their integration in OR-PAM systems is attractive for applicaitons in vascular biology research, such as spatially resolved blood flow and oxygenation measurements in studies of neovascularization, where a combination of high acoustic sensitivity, spatial resolution, and imaging frame rate is required [6,9]. Such a system was first described by by Hajireza et al. [45]. The sensor, which consisted of dielectric mirrors and a polymer film spacer coated onto a glass substrate, had a -3 dB bandwidth of 18 MHz and was interrogated by a C-band continuous wave (CW) laser. The *in vivo* imaging capabilities were demonstrated in a chicken embryo.

In this study, a backward-mode OR-PAM system based on a planar FP polymer film sensor was developed. The sensor was deposited onto a polymer substrate to achieve minimal acoustic impedance mismatch and hence a broadband, uniform acoustic detection bandwidth. The dielectric mirrors were designed for high transmission in both the visible (450 nm – 630 nm) and the near-infrared wavelength region to allow the backward mode excitation and detection of PA signals and, potentially, multiphoton fluorescence signals. High mirror reflectivity around 780 nm yielded a high sensor finesse, and the homogeneity of the optical thickness of the FP etalon was sufficient to enable fast signal acquisition without bias tuning over a field of view (FOV) of several mm^2^. By separating the focal planes of the coaxially aligned excitation and sensor interrogation beams, minimal source-detector distances, and hence minimal losses due to geometric spreading of the PA waves, were achieved. In addition, coaxial alignment of the beams allowed a direct conversion of time-resolved PA waves into depth profiles of initial pressure. High frame rates were achieved by scanning the coaligned beams continuously at high pulse repetition frequencies across the sample. The capabilities of the FP based OR-PAM system are demonstrated by imaging a phantom and zebrafish embryos in an initial *in vivo* study. The optical design of the sensor offers the opportunity to combine OR-PAM with other technologies, such as optical coherence tomography, confocal or multiphoton optical microscopy, to enable multi-modal imaging in a single instrument in the future.

## 2 Materials and methods

### 2.1 Optical setup

**Fig. 1:**
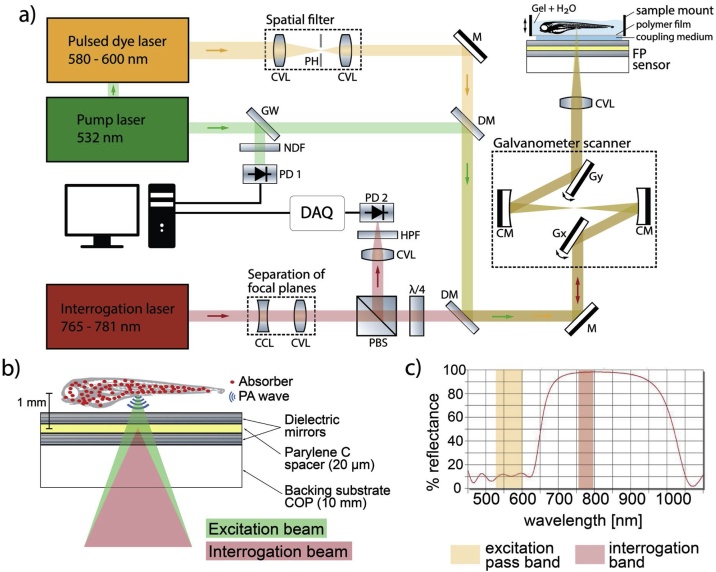
Experimental setup of the backward-mode OR-PAM system based on a planar Fabry-Pérot (FP) ultrasound sensor. **a** Schematic of the OR-PAM system. **b** Schematic of the FP sensor. **c** Reflectance spectrum of the dielectric mirrors of the FP sensor. CCL = Concave Lens, CM = Concave Mirror, CVL = Convex Lens, DAQ = Data Acquisition, DM = Dichroic Mirror, FP = Fabry-Pérot, GW = Glass Window, Gx = X-Galvanometer mirror, Gy = Y-Galvanometer mirror, HPF = High-Pass Filter, M = Mirror, NDF = Neutral Density Filter, PBS = Polarizing Beamsplitter, PD = Photodiode, PH = Pinhole, λ/4 = Quarter-Wave Plate

The setup of the backward-mode OR-PAM system was based on a planar FP sensor as shown in Fig. 1a. A wavelength-tunable ns-pulsed dye laser system with a maximum repetition rate of 600 kHz and a variable pulse duration of 3 ns–20 ns was used to generate PA signals. The laser system provided separate outputs from the pump laser (532 nm, VGEN-G-20, Newport) and the dye laser (580 nm–600 nm, Credo Dye, Sirah). To improve the beam quality of the dye laser, its output was guided through a spatial filter, *i.e.* a pinhole of 30 μm diameter (Thorlabs), using two lenses (f = 75 mm, Thorlabs) to obtain a collimated beam with a near-Gaussian intensity profile. A small portion of the excitation light was guided towards an Si photodiode (Hamamatsu, PD 1 in Fig. 1a) for triggering and monitoring the pulse energy.

PA waves were detected using a planar FP ultrasound sensor, which consisted of a 20 μm polymer spacer (Parylene C) sandwiched between two dielectric mirrors (ZnS and Na_3_AlF_6_) deposited on a cyclo-olefin polymer (COP) backing substrate of 1 cm thickness (Fig. 1b). The mirrors formed an interferometer etalon and were designed to be highly reflective (95–98 %) in the interrogation band (750−800 nm) and highly transparent at the main OR-PAM excitation wavelengths (532 nm, 580−600 nm) and the near-infrared wavelengths that are commonly used for multi-photon microscopy of fluorescent proteins (1000−1100 nm) (Fig. 1c). The comparatively high transmission at near-infrared wavelengths may allow a combination of OR-PAM and purely optical microscopy in the future.

The FP sensor was illuminated by the output of a CW tunable interrogation laser (765−781 nm, New Focus Velocity, Newport) as illustrated in Fig. 1b. Its transduction mechanism relied on the acoustic modulation of the optical thickness of the spacer material caused by an incident PA wave. The thickness modulation changes the phase difference between the optical reflections from the mirrors. This, in turn, modulates the reflected optical power detected using a photodiode (Hamamatsu, PD 2 in Fig. 1a). The interferometer transfer function (ITF) was obtained by measuring the reflected optical power as a function of wavelength. A typical ITF shows several interference fringes over the entire interrogation wavelength range. The maximum optical phase sensitivity and hence acoustic sensitivity is found at wavelengths where the ITF slope is at a maximum [46]. For ultrasound detection, the working point of the interferometer is set such that the interrogation wavelength coincides with the steepest slope of the ITF.

To minimize the source-detector distance, the interrogation and excitation beams were coaxially aligned. An achromatic doublet (f = 50 mm, Edmund Optics) was used to focus the beams. While the excitation light was focussed into the sample, additional lenses were used to adjust the divergence of the interrogation beam such that its focus coincided with the plane of the FP sensor. The specifications of the lenses were obtained using optics design software. The focal planes of the excitation and interrogation beams were separated by approximately 1 mm. The interrogation light reflected from the sensor was guided to an Si photodiode using a quarter-wave plate (Thorlabs), a polarizing beam-splitter (Thorlabs), and a high pass filter (Semrock, 715 nm cut-off) to block the excitation light. The coaligned beams were scanned across the sensor using galvanometer mirrors driven by a multifunction I/O device (PXI-6229, National Instruments). Concave mirrors were used to pivot the beams pivot on a point on the optical axis. This ensured near-constant focal spot sizes and minimal aberrations over the entire field of view (FOV). The time-course of the PA signals and the trigger pulses were recorded using a digital oscilloscope (PXIe-5160, National Instruments). The samples were placed in a holder with a transparent polymer film window positioned above the FP sensor using a translation stage. Water or aqueous gel provided acoustic coupling.

### 2.2 Scanning modes

The system could be operated in a comparatively slow raster-scanning mode or a fast continuous-scanning mode. In raster-scanning mode, the galvanometer mirrors translated the coaligned beams across the FOV in a stepwise manner. At each position, the interrogation wavelength was adjusted to compensate for minor variations in the optical thickness of the FP sensor and to maximize the acoustic sensitivity. The excitation pulse then generated a PA wave in the sample, the time-course of which was recorded using the optical interrogation of the FP sensor. As a result, the scan time for a FOV of (1 × 1) mm^2^ using a step size of 4 μm was approximately 40 s. PA images were generated by converting time to distance using the speed of sound in water (c_s_ = 1481 m/s). 3D volume-rendered images were obtained using Amira.

To address the limitations of the slow raster-scanning mode, a fast signal acquisition method based on continuously moving galvanometer mirrors and data streaming was implemented [36]. While the excitation and interrogation beams were translated continuously across the FOV, time-resolved PA signals (using PD 2 in Fig. 1a) and the time-trace of the excitation pulses (using PD 1 in Fig. 1a) were recorded. The latter was used to format the streamed data set into an *x-y-t* image data set by matching the time points of the excitation pulses with the corresponding PA signals and galvanometer positions. The recorded signals were processed by applying a Hilbert transform. To achieve the necessary data transfer rates, data streaming was used. This also limited the maximum temporal sampling rate of the system to 50 MS/s per channel.

During continuous-scanning, the interrogation wavelength remained constant. To ensure constant acoustic sensitivity across the FOV, a region of the FP sensor with high homogeneity of optical thickness was selected. The image acquisition speed of the continuous-scanning method was determined by the pulse repetition rate (PRR) of the excitation laser. For example, for a FOV of (1 × 1) mm^2^ and (250 × 250) pixels (corresponding to an effective step size of 4 μm) and a PRR of 100 kHz, the image acquisition time was approximately 0.6 s. The excitation beam moved by 4 nm during a 10 ns pulse. Until a signal reached the detector from 1 mm depth, the interrogation beam moved by 27 μm.

### 2.3 System characterization and OR-PAM of phantoms

The acoustic frequency response of the FP sensor was modeled as described in [46] with the appropriate density and speed of sound values for the backing substrate and the spacer material. The lateral resolution of the system in raster-scanning mode was determined as described in [47]. To determine the lateral resolution in continuous-scanning mode, a negative USAF resolution test target (Thorlabs) was imaged at an excitation wavelength of 532 nm using 50 kHz over a FOV of (1 × 1) mm^2^ with an effective step size of 2 μm. A Boltzmann function was fitted to a profile of the image intensity across the edge of several resolution lines in the target to determine edge-spread functions (ESF). Line-spread functions (LSF) were obtained by taking the first derivative of the ESFs. A Gaussian function was then fitted to each LSF to arrive at the definition for the lateral resolution, *i.e.* the full-width at half-maximum (FWHM). The FWHMs of all LSF fits were averaged to obtain a final value for the lateral resolution.

To demonstrate the structural imaging capabilities of the system, a black leaf skeleton was imaged. Following excitation at 532 nm and 590 nm at a PRR of 20 kHz and pulse energies down to 40 nJ, PA signals were acquired. For this pulse energy, the fluence at the focus was estimated to be 100 mJ/cm^2^, which is much lower than in other *in vivo* OR-PAM applications [9,24,36,37]. In raster-scanning mode, the galvanometer mirrors had a step size of 10 μm and signals were averaged 20 times. For a FOV of (3 × 3) mm^2^, the total image acquisition time was 22 min. In continuous-scanning mode, 50 kHz and 100 kHz PRR were used to record PA signals without signal averaging, and the effective step size was 4 μm. For a PRR of 50 kHz and a FOV of (2 × 2) mm^2^, the image acquisition time was 2.5 s.

The signal-to-noise (SNR) of OR-PAM images was defined as the ratio of the PA amplitude over the root mean squared (RMS) noise, where the peak-to-peak noise corresponded to six times the RMS value.

### 2.4 *In vivo* OR-PAM of zebrafish embryos

Zebrafish embryos (strain AB/TL, 4 days post fertilization (dpf)) were anesthetized in 0.014 % tricaine (Tricaine-Pharmaq, 1000 mg/g, PHARMAQ Limited). For imaging, the embryos were mounted in low melting point agarose gel (Invitrogen) containing 0.014 % tricaine, which was placed on a transparent foil within a mechanical mount, and bathed in E3 media containing 0.014 % tricaine. Zebrafish growing, breeding, and handling complied with the regulations of the Animal Welfare Committee at the MDC and with FELASA guidelines [48]. The embryos were positioned approximately 1 mm above the FP sensor using translation stages. OR-PAM data sets were acquired in raster-scanning mode at an excitation wavelength of 532 nm and a PRR of 20 kHz across a FOV of (1.2 × 3.8) mm^2^ with 8 μm step-size. The pulse energy was below 200 nJ. PA signals were averaged 20 times for each pixel, resulting in a total image acquisition time of 17 min.

## 3 Results

### 3.1 System characterisation

**Fig. 2:**
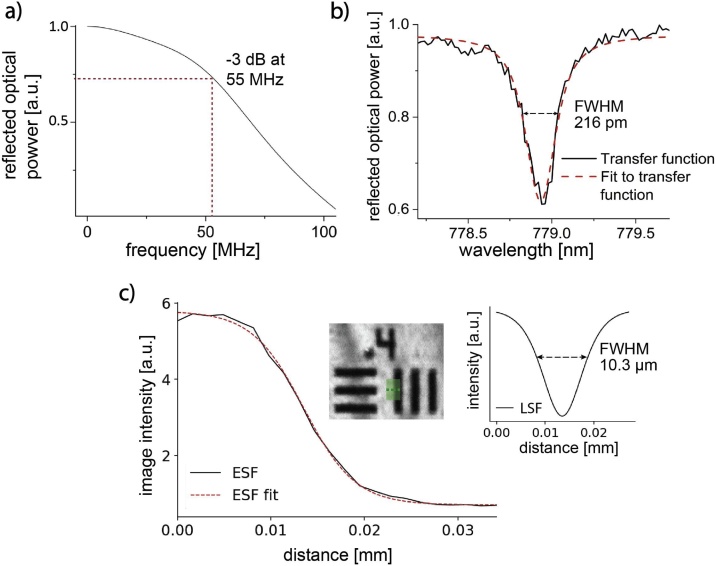
Scanner characterization. **a** Frequency response of an FP sensor with a dielectric mirror reflectivity of 98 % and spacer thickness 20 μm (using the model published in [28]). **b** Typical interferometer transfer function (ITF). **c** Edge-spread function (ESF) corresponding to the averaged image intensity along the green line in the continuous-scanning OR-PAM image of a resolution target shown in the left inset. The FWHM of the line-spread function (LSF, right inset) was obtained as a measure of the lateral resolution. On average, it was found to be (8.1 ± 1.4) μm in the raster-scanning and (11.8 ± 1.7) μm in the continuous-scanning mode.

The frequency response of an FP sensor with a 20 μm spacer is shown in Fig. 2a. It was found to be broadband, with a uniform response from dc up to the -3 dB value at 55 MHz. Fig. 2b shows an example of a measured ITF. Across the scan area, ITFs showed resonances with an average FWHM of (336 ± 120) pm and a free spectral range of approximately 8 nm. The lateral resolution obtained from measurements of the ESF in raster-scanning mode was found to be (8.1 ± 1.4) μm (mean ± standard deviation, n = 3, with a maximum resolution of 6.48 μm). This is in good agreement with the diffraction-limited spot size of the lens of approximately 6.6 μm. In continuous-scanning mode, the lateral resolution was found to be (11.8 ± 1.7) μm (mean ± standard deviation, n = 8, with a maximum resolution of 9.36 μm, Fig. 2c). The vertical resolution is estimated to be 15 μm given the typical excitation pulse width of 10 ns and the speed of sound in water. Since the excitation laser offers pulse durations down to 3 ns, the vertical resolution may be further increased provided the bandwidth of the detector can also be broadened. For example, using an FP spacer thickness of 10 μm would provide a bandwidth of up to 100 MHz, which would result in a potential vertical resolution of approximately 8 μm. In continuous-scanning mode, the vertical resolution was limited to 30 μm due to the maximum sampling frequency imposed by the data streaming rate of the current system architecture (50 MS/s). Given that systems with substantially higher sampling rates are readily available, the current vertical resolution does not represent a fundamental limitation.

### 3.2 OR-PAM of a leaf skeleton phantom

**Fig. 3:**
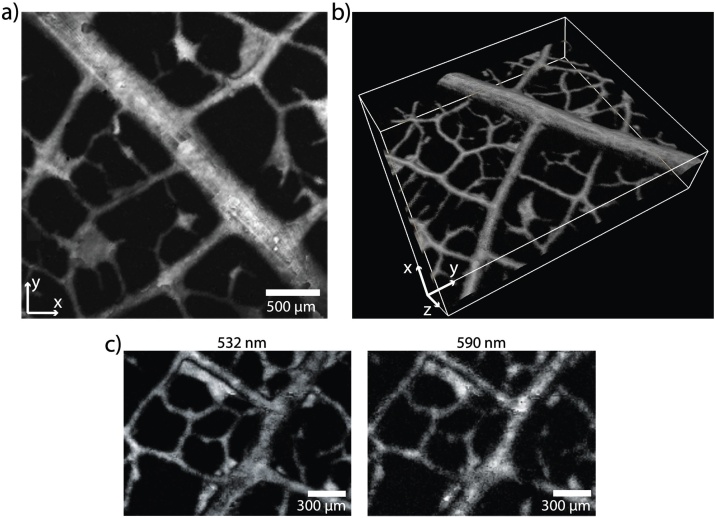
OR-PAM image of a leaf skeleton phantom acquired in raster-scanning mode. **a** x-y-maximum intensity projections (MIP) of the image. **b** Volume-rendered 3D image of an OR-PAM data set. **c** Comparison of OR-PAM images of the same leaf skeleton phantom obtained at excitation wavelengths of 532 nm and 590 nm, respectively.

Fig. 3 shows 2D maximum intensity projections (MIP) and a 3D volume-rendered image dataset of a black leaf skeleton phantom acquired in raster-scanning mode using the Fabry-Pérot OR-PAM system. Due to the coaxial alignment of the excitation and interrogation beams, depth-resolved information is obtained from the time-course of the PA signals as evidenced by the *x-z-* and *y-z-*MIPs (Fig. 3a) and the visualization of the curvature and contours of the leaf (Fig. 3b).

To demonstrate multiwavelength imaging capabilities, which will allow the recovery of functional hemodynamic parameters in future *in vivo* applications of the scanner, images of the leaf phantom were acquired at 532 nm and 590 nm (Fig. 3c). While the morphology is visible in both images, the spatial resolution and SNR of the image acquired at the dye laser wavelength of 590 nm are lower compared to that acquired using the output of the fiber-based pump laser at 532 nm. The reason for the discrepancy is primarily due to the poor beam quality of the dye laser, which the spatial filter could not sufficiently remedy.

**Fig. 4:**
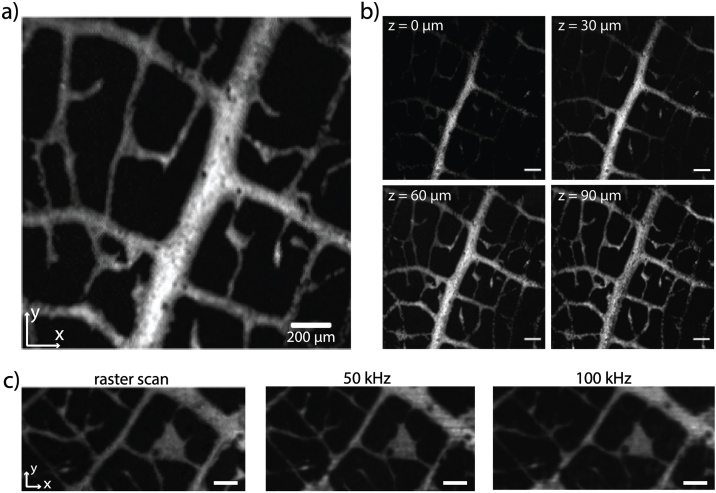
OR-PAM image of a leaf skeleton phantom acquired in continuous-scanning mode. The scale bar represents 200 μm. **a** xy-MIP of the Hilbert transform of the PA signals. **b** z-slices at different depths of the data set shown in (a). **c** Comparison of images acquired in raster-scanning mode, at a PRR of 50 kHz, and at 100 kHz in continuous-scanning mode.

The envisaged application of the scanner in functional imaging studies of vascular development requires the system to acquire images at frame rates greater than 20 Hz [10]. To test the performance of the system, the continuous-scanning mode was used on a leaf skeleton phantom. For a PRR of 100 kHz and a FOV of (2 × 2) mm^2^ (500 × 500 pixels), a frame rate of 0.4 Hz was obtained. For a FOV of (300 × 200) μm^2^, which is typically used to image the zebrafish embryo trunk, frame rates which would fulfill the requirements for measuring blood flow are possible when operating the galvanometer scanner at 1 kHz in the fast scanning axis [49]. Fig. 4a and b illustrate the depth resolution of the system. Fig. 4a shows an *x-y-*MIP of a PA image of the leaf phantom while Fig. 4b shows cross-sectional layers at different depths. The dark circular regions along the stem appear in the image due to local inhomogeneities in the spacer thickness that have arisen from contamination during sensor fabrication. A comparison of the same FOV obtained in raster-scanning mode and continuous-scanning mode at two different PRRs is presented in Fig. 4c. While the SNR in the raster-scanning image amounted to 125, it was 58 in the 50 kHz continuous-scanning image and 54 in the 100 kHz image. As expected, the contrast is reduced for the fast image acquisitions as the interrogation wavelength was kept constant but the image quality is sufficient to facilitate high frame rate imaging.

### 3.3 *In vivo* imaging of zebrafish embryo

**Fig. 5:**
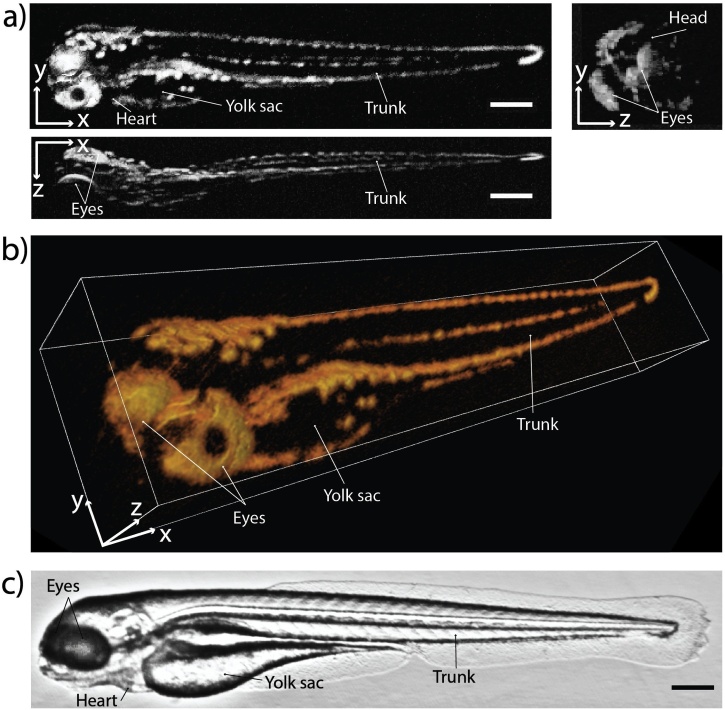
*In vivo* OR-PAM of a zebrafish embryo (4 dpf) acquired in raster-scanning mode. **a***x-y-*, *x-z-*, and *y-z-*MIPs of the image. **b** Volume-rendered 3D image of the same data set shown in (a). **c** Zebrafish anatomy in a bright-field microscopy image for reference. Scale bars represent 300 μm.

In an initial *in vivo* experiment, OR-PAM images of zebrafish embryos were obtained. Fig. 5 shows an example in 2D and 3D representation. The eyes, yolk sac, trunk, and pigmented skin are resolved. While hemoglobin contributed to the PA contrast, the strong absorption of melanin is likely dominant in this image. A bright-field image of a zebrafish at the same age with labeled features is shown for reference (Fig. 5c).

## 4 Discussion

In this work, an OR-PAM system based on a planar Fabry-Pérot sensor was developed for multimodal functional imaging applications in large model organisms. The design considerations included backward-mode operation, multiwavelength excitation, fast PA signal acquisition for high frame rates, high acoustic sensitivity, broad detection bandwidth, and the option to integrate the FP-based microscope with other optical imaging modalities such as multi-photon fluorescence microscopy (MPM) or optical coherence tomography (OCT).

Multiwavelength backward-mode imaging was demonstrated in leaf skeleton phantoms and in zebrafish embryos *in vivo*. The difference in spatial resolution and contrast obtained in phantom measurements is explained by the larger beam divergence of the dye laser (590 nm) compared to that of the 532 nm (pump) fiber laser. Optimal beam parameters may be obtained in the future by integrating other excitation sources, such as Raman lasers, which have been shown to produce Gaussian beams at a variety of excitation wavelengths suitable for functional imaging [50,51].

To achieve high frame rates, a method for fast signal acquisition based on the continuous movement of the galvanometer mirrors and data-streaming was implemented. While raster-scanning provides optimum bias at each scan position by tuning the interrogation wavelength, it is also slower due to the settling times of the galvanometer mirrors and the interrogation laser. For settings suitable for imaging of functional hemodynamic parameters in the zebrafish embryo, *e.g.* a FOV of (300 × 200) μm^2^ and a step size of 4 μm, raster-scanning would result in a frame rate of 0.4 Hz, which is below frame rates of >20 Hz required for imaging hemodynamic processes. By comparison, the continuous-scanning mode is 60 times faster for the same FOV (with a PRR of 100 kHz) and provides frame rates of more than 25 Hz, which is sufficiently fast to resolve blood flow in small animals [10]. While the planar architecture of the sensor together with the coaxial alignment of the interrogation and excitation beams allows for minimal source-detector distances, and thus potentially high acoustic detection sensitivity, SNR was found to be affected by the scanning mode and the pulse repetition frequency. For example, continuous-scanning phantom measurements yielded an SNR that was a factor of two lower compared to measurements obtained in raster-scanning mode. The lower SNR was expected as the interrogation wavelength was fixed in the continuous-scanning mode and could not be tuned to optimal acoustic sensitivity for each sensor region. Also, the vertical resolution in the continuous-scanning mode was limited by the sampling rate of the current data acquisition system. However, this can be readily increased to cover the full acoustic detection bandwidth of the FP sensor (*e.g.* ∼8 μm for a sensor with a bandwidth of 100 MHz and a pulse duration of 3 ns). The continuous-scanning mode also requires an FP sensor with high homogeneity of optical thickness over the entire FOV to allow single wavelength interrogation. Thickness inhomogeneities are likely to have contributed to lower SNR observed in the continuous-scanning mode.

While the performance of the sensors used in this study was affected by inhomogeneities in spacer thickness and contaminations (evident as dark regions or regions of lower intensity in the continuous-scanning images), both can be improved by optimizing the fabrication process. The frequency response of the FP sensors used in this study is uniform up to 55 MHz (-3 dB). It may be increased in the future by reducing the spacer thickness (*e.g.* 10 μm for a bandwidth of up to 100 MHz at -3 dB) to provide an optimal match to the frequency content of typical OR-PAM signals [52]. The acoustic sensitivity may also be increased by optimizing the mirror reflectivity and the mechanical properties of the spacer materials.

The system design also offers the potential to combine OR-PAM with MPM as the mirrors of the FP sensor are designed to be transparent across excitation wavelength ranges relevant to both modalities. This may enable inherently co-registered functional and molecular high-resolution imaging of the vasculature in large organisms *in vivo*, such as the longitudinal study of vascularisation in the mouse brain.

## 5 Conclusion

A backward-mode OR-PAM system based on a planar FP ultrasound sensor is described. The system is designed to meet the requirements for *in vivo* functional OR-PAM applications, such as fast image acquisition, broadband ultrasound detection, and minimal source-detector distances for high acoustic sensitivity. The novel planar FP sensor features a high finesse and a high spacer homogeneity over a FOV of several mm^2^. To facilitate fast image acquisition, a continuous-scanning mode can be used to acquire 3D-image datasets at frame rates of 25 Hz over FOV typically used for imaging the zebrafish trunk. The capabilities of the system were demonstrated by imaging leaf skeleton phantoms and living zebrafish embryos. The FP-based OR-PAM scanner has the potential to be combined with other microscopy modalities, such as multi-photon microscopy, to provide single-cell resolution spatial imaging and functional OR-PAM of the vasculature *in vivo*.

## Author statement

**Elisabeth Baumann**: Investigation, Validation, Software, Writing - Original Draft Preparation. **Ulrike Pohle**: Investigation, Validation, Writing - Original Draft Preparation. **Edward Zhang**: Methodology, Resources. **Thomas Allen**: Software, Writing – Review & Editing. **Claus Villringer**: Resources. **Silvio Pulwer**: Resources. **Holger Gerhardt**: Conceptualisation, Supervision, Funding Acquisition. **Jan Laufer**: Conceptualisation, Funding Acquisition, Project Administration, Supervision, Writing – Review & Editing.

## Funding information

This project was funded by the Deutsche Forschungsgemeinschaft, DFG (LA3273/3-1, GE 2154/1-1).

## Declaration of Competing Interest

The authors declare that there are no conflicts of interest.
